# Stimulation parameters for directional vagus nerve stimulation

**DOI:** 10.1186/s42234-023-00117-2

**Published:** 2023-07-18

**Authors:** Joel Villalobos, Sophie C. Payne, Glenn M. Ward, Sofianos Andrikopoulos, Tomoko Hyakumura, Richard J. MacIsaac, James B. Fallon

**Affiliations:** 1 Bionics Institute, East Melbourne, Vic Australia; 2 Department of Medical Bionics, University of Melbourne, Parkville, Vic Australia; 3 Department of Endocrinology and Diabetes, St Vincent’s Hospital Melbourne, Fitzroy, Vic Australia; 4 Department of Medicine, St Vincent’s Hospital Melbourne, University of Melbourne, Fitzroy, Vic Australia; 5 Australian Centre for Accelerating Diabetes Innovations, University of Melbourne, Parkville, Australia; 6 Department of Medicine (Austin Health), University of Melbourne, Heidelberg, Vic Australia; 7 Australian Diabetes Society, Sydney, NSW Australia

**Keywords:** Peripheral nerve stimulation, Metabolic disease, Medical devices, Bioelectric medicine, Nerve blocking

## Abstract

**Background:**

Autonomic nerve stimulation is used as a treatment for a growing number of diseases. We have previously demonstrated that application of efferent vagus nerve stimulation (eVNS) has promising glucose lowering effects in a rat model of type 2 diabetes. This paradigm combines high frequency pulsatile stimulation to block nerve activation in the afferent direction with low frequency stimulation to activate the efferent nerve section. In this study we explored the effects of the parameters for nerve blocking on the ability to inhibit nerve activation in the afferent direction. The overarching aim is to establish a blocking stimulation strategy that could be applied using commercially available implantable pulse generators used in the clinic.

**Methods:**

Male rats (*n* = 20) had the anterior abdominal vagus nerve implanted with a multi-electrode cuff. Evoked compound action potentials (ECAP) were recorded at the proximal end of the electrode cuff. The efficacy of high frequency stimulation to block the afferent ECAP was assessed by changes in the threshold and saturation level of the response. Blocking frequency and duty cycle of the blocking pulses were varied while maintaining a constant 4 mA current amplitude.

**Results:**

During application of blocking at lower frequencies (≤ 4 kHz), the ECAP threshold increased (ANOVA, *p* < 0.001) and saturation level decreased (*p* < 0.001). Application of higher duty cycles (> 70%) led to an increase in evoked neural response threshold (*p* < 0.001) and a decrease in saturation level (*p* < 0.001). During the application of a constant pulse width and frequency (1 or 1.6 kHz, > 70% duty cycle), the charge delivered per pulse had a significant influence on the magnitude of the block (ANOVA, *p* = 0.003), and was focal (< 2 mm range).

**Conclusions:**

This study has determined the range of frequencies, duty cycles and currents of high frequency stimulation that generate an efficacious, focal axonal block of a predominantly C-fiber tract. These findings could have potential application for the treatment of type 2 diabetes.

## Introduction

Peripheral nerve stimulation is a growing field in which neuromodulation technology is used as a clinical therapy for epilepsy, depression, obesity, heart failure and shows promise for the treatment of rheumatoid arthritis and inflammatory bowel disease (Naufel et al. 2020; Cracchiolo et al. 2021). The premise of electrical neuromodulation technology is to take advantage of natural neural innervation patterns that target systems and organs for modulating specific physiological functions (Schulkin and Sterling 2019). For example, the vagus nerve has significant roles in the regulation of energy metabolism, food intake and glycemia as the nerve has a major role in the control of pancreatic hormonal secretions (Waise et al. 2018). It has been known for decades that vagus nerve stimulation modifies secretion of insulin and glucagon, the two opposing hormones that control the levels of glucose in the blood (Ionescu et al. 1983; Ahren et al. 1986; Nishi et al. 1987; Peitl et al. 2005; Babic et al. 2012). Recent preclinical (Meyers et al. 2016; Malbert 2018; Yin et al. 2019) and human (Shikora et al. 2013) studies indicate that neuromodulation of the vagus nerve could have applications for the treatment of type 2 diabetes (Guemes Gonzalez et al. 2020). Our own work has utilized an emerging type of neuromodulation that applies an electrical block in the presence of activating stimuli to cause preferential activation of the distal (efferent) vagus nerve pathway (Payne et al. 2020, 2022). Application of efferent vagus nerve stimulation (eVNS) within the abdomen reduced the glycemic response during an oral glucose test in a diabetic rat model (Payne et al. 2022). Furthermore, eVNS was safe and well tolerated in rats over the 5-week implantation testing period (Payne et al. 2022). Although promising, the high frequency blocking stimulation parameters used (26 kHz) are currently outside the range of what can be easily delivered with commercially available implantable pulse generators (Payne et al. 2022). Therefore, the overall aim of this study is to establish a range of lower frequency blocking stimulation parameters that could be applied using commercially available implantable pulse generators used in the clinic. This is an important next step towards clinical translation.

Preferential activation of either the distal (efferent) or proximal (afferent) nerve pathway requires a safe, reversible electrical block of nerve signaling in the presence of activating stimuli (Kilgore and Bhadra 2004a). Such directional stimulation could also have application in reducing unwanted activation of fibers that cause undesirable off-target effects during cervical vagus nerve stimulation (Grill and Mortimer 1995; Ahmed et al. 2022). Bipolar nerve stimulation depolarises the axonal membrane and the generation of action potentials near the cathode. When the elicited action potentials reach sufficiently hyperpolarized sections of axon near the anode, conduction of some fibres is blocked (Kwon and Jae 1994). In this way, cathode caudad polarity favours the activation of efferent fibres, whereas cathode cephalad favours the activation of afferent fibres. Although anodal block to induce directional nerve signalling of the cervical vagus nerve has been used clinically for the treatment of epilepsy (Arle et al. 2016) and heart failure (Premchand et al. 2014), it is often unreliable and produces only a partial block (Dreyer et al. 1993). There is growing interest in the use of a charge-balanced kilohertz frequency alternating current (KHFAC: 1 – 100 kHz) to generate a rapid, focal and reversible block of action potentials from all fibre types (A- to C) (Kilgore and Bhadra 2004a; Green et al. 2022). The KHFAC block can be combined with activating stimuli applied to distal or proximal electrodes to activate the afferent or efferent nerve bundle, respectively (Patel and Butera 2015; Patel et al. 2017; Payne et al. 2020, 2022). Our KHFAC blocking strategy (26 kHz) is well tolerated in awake animals and does not damage the nerve after 5 weeks of stimulation (1 h/day, 13–27 h total stimulation time) (Payne et al. 2022). As such, it is the first aim of this study to use electrophysiological methods to assess the capacity of lower frequency blocking strategies.

In our previous work, we used our 3-pair electrode array to record evoked potentials using the outer electrode pairs while applying KHFAC blocking to the middle electrode pair to determine the efficacy of blocking to inhibit activity (Payne et al. 2020, 2022). Electrophysiological confirmation of the efficacy of blocking has not often occurred in either preclinical or clinical studies (Tweden et al. 2006b, 2006a; Camilleri et al. 2009). Assessing the efficacy of blocking is an essential feature for our directional stimulation strategy as the block is compromised when higher amounts of current are applied to the distal electrode pair. Determining the therapeutic (blocking) window, which is the range of current levels at which the directionality of the activating stimuli is maintained, is critical. Therefore, the second aim is to improve extraction of the evoked neural signal from the overwhelming artifact resulting from the high frequency blocking.

**Fig. 1:**
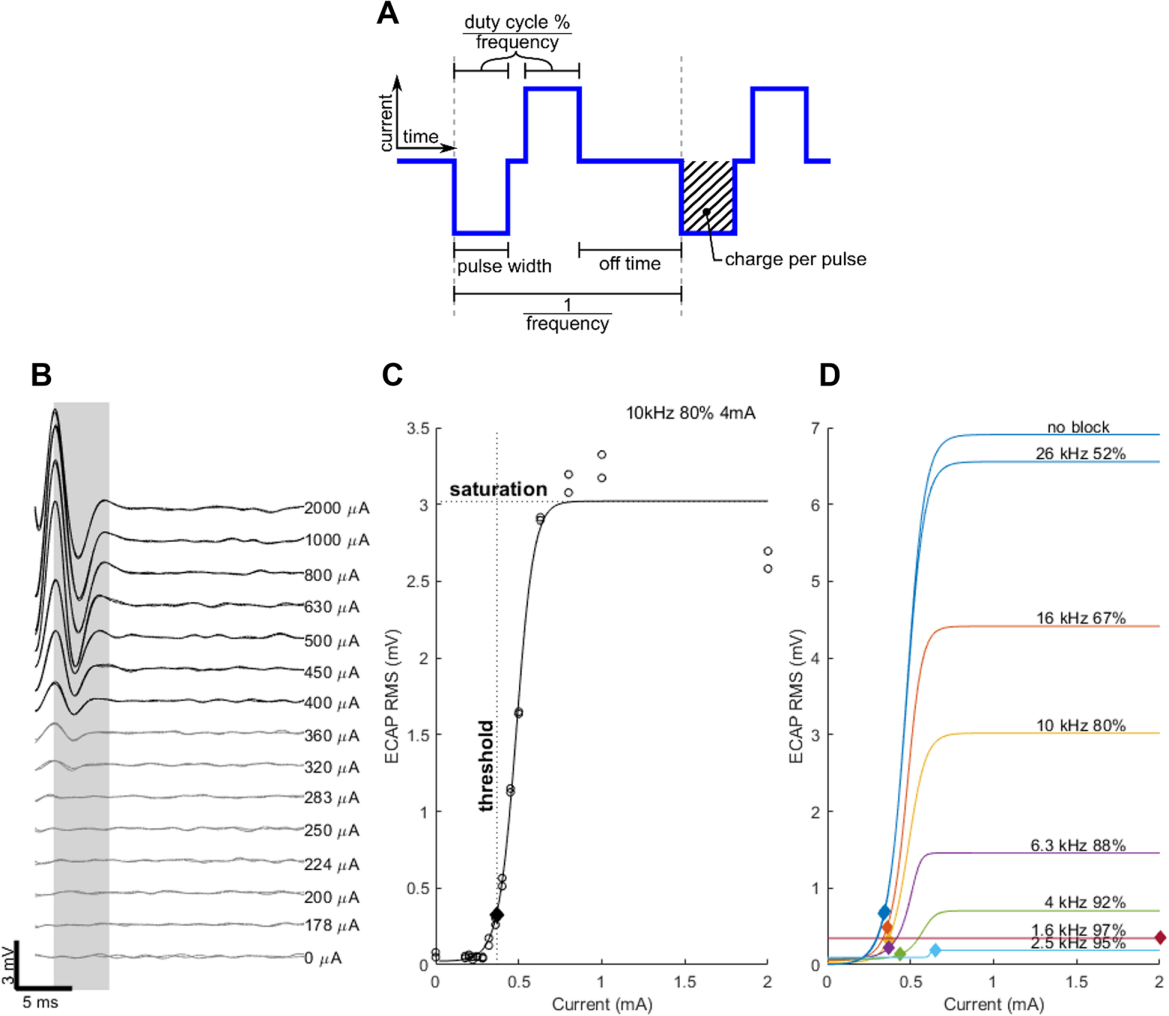
Electrophysiology methods.** A** Diagram defining the stimulation pulse timing parameters used for analysis. **B** Example of evoked compound action potentials (ECAPs) in rat vagus nerve quantified at increasing activation current amplitude in the period 3–9 ms post-stimulus (shaded area). This ECAP was recorded on the rostral electrode pair during 10 kHz, 80% duty cycle and 4 mA current blocking stimuli applied to the middle electrode pair. **C** The resultant ECAP RMS response (generated from the shaded area in B) to stimulus current was fit with a sigmoidal curve to identify response saturation level and threshold. **D** ECAP responses (threshold and saturation) were compared at various blocking frequencies (1.6 – 26 kHz) and duty cycles (1 / frequency—18 μs) / 2, displayed as %)

This work in anaesthetized rats used electrophysiological recording techniques to understand the link between frequency of the pulsatile stimulation strategy and the ability to block the nerve conduction. The driving hypothesis for the studies described here was that there is an optimal stimulation range that yields an increased therapeutic window, so we explored the efficacy of pulsatile (Fig. 1A) blocking at a range of frequencies below the 26 kHz used previously (Payne et al. 2022) and using different pulse widths. Furthermore, a novel aspect of the studies described was the use of improved extraction of the evoked neural signal using a cascade filter to overcome the overwhelming artifact noise resulting from high frequency blocking.

## Methods

### Animals and anesthesia

All experiments used normal male Sprague–Dawley rats (*n* = 20 rats at 8–10 weeks, Animal Resource Centre, Western Australia). Procedures were approved by St Vincent’s Hospital Animal Research Ethics Committee (Project/approval number: 023–21) and complied with the Australian Code for the Care and Use of Animals for Scientific Purposes (National Health and Medical Research Council of Australia) and the Prevention of Cruelty to Animals (1986) Act. Animals were kept on a 12 h light/dark cycle and allowed access to standard chow and water ad libitum. Rats were fasted overnight (14–16 h) and anaesthetized (induction: 3%, maintenance during surgery 2–2.5%, maintenance during electrophysiology testing: 1.5–2% isoflurane, in oxygen flowing at 1 L/min) prior to the terminal surgical procedure. Breathing rate was maintained between 45 and 60 breaths per minute for the duration of the non-recovery experiment by adjusting the level of isoflurane. At the conclusion of the experiment, rats were euthanized (300 mg/kg Lethabarb, intravenous injection).

### Vagus nerve electrode array

The vagus nerve electrode cuff contained platinum electrodes in a silicone elastomer substrate. The initial experiments used a previously developed array with 3-pairs of electrodes (*n* = 15) in which the distance between electrode pairs was 3.4 mm, similar to that used previously (Payne et al. 2020, 2022). A new prototype array that had 4-pairs of electrodes was used in later experiments (*n* = 5) in which the distance between electrode pairs was 2.22 mm. For both array designs platinum electrodes had an exposed surface area of 0.39 mm^2^ and were arranged in pairs, on opposite sides of the cuff. A rectangular-section lumen (0.55 mm × 0.2 mm) traversed the cuff for housing the abdominal vagus nerve. The silicone cuff was sutured closed to prevent the nerve from migrating away from the electrode interface. A silicone suturing tab on the lead was used to anchor the array to the esophagus to provide mechanical stability, which was attached to the helical cable that ran to an external connector.

### Electrode implant surgery

As described previously (Payne et al. 2020, 2022), the ventral abdominal midline was incised and the ventral esophagus and sub-diaphragmatic anterior abdominal branch of the vagus nerve exposed. The nerve was carefully dissected away from the esophagus and the array implanted rostral to the hepatic and celiac branches of the vagus (Payne et al. 2019). The implant was sutured to the esophagus for stability. The cable exited through the abdominal incision and the wound was held closed during the experiment.

### Electrophysiological recordings and stimulation

*Common-ground impedance*: Common-ground impedance was used to test functionality of electrodes by measuring the impedance from voltage transients to current pulses (25 µs, 100 µA) from each electrode against all others as return using a custom-made external stimulator and visualized using IGOR Pro-8 (Fallon et al., 2018) (Payne et al. 2022).

*Evoked compound action potentials (ECAPs)*: were recorded to determine the amplitude of electrically-evoked neural responses. ECAPs were generated by delivering bipolar stimulation to the distal (caudal) electrode pair (200 µs; 15 Hz; 0–2 mA in logarithmic steps) using a custom-made external stimulator (Fallon et al., 2018). Further smaller steps of stimulus current (0.5 dB or 1 dB) near the provisional ECAP threshold were generated to improve the accuracy of the neural threshold. Recording from the proximal (rostral) pair (averaged over 100 pulses), which were sampled at a rate of 200 kHz using a data acquisition device (USB-6210, National Instruments) (Payne et al. 2019; Fallon and Payne 2020). The signals were filtered in two stages to reduce the artifact from blocking stimuli. First all the frequencies above 900 Hz were blanked in Fourier space and afterwards a time-domain digital filter was applied with a band pass of 20–800 Hz. The threshold of evoked neural responses was visualized using IGOR Pro-8. The provisional ECAP threshold during the experiment was defined as the perceived inflection point where stimulus intensity producing a monotonically increasing response amplitude within a post-stimulus latency window of 3–9 ms (Fig. 1). This latency corresponded to expected conduction velocities within the range of C-fiber responses (Castoro et al. 2011). In all analyzed experiments the stimulation current delivered to the distal electrode pair was supra-threshold.

*Directional efferent* s*timulation*: Directional efferent stimulation, referred to as ‘eVNS’, was simultaneously applied high frequency pulses to the central electrode pair and 15 Hz, 200 µs pulses to the distal electrode pair (Payne et al. 2022). A custom-made external stimulator was used to deliver biphasic current pulses (Fallon et al., 2018).

*Pulsatile blocking stimulation parameters*: The high frequency pulses (symmetric biphasic charge balanced pulses) were delivered at a constant amplitude of 4 mA but changed frequency in logarithmic steps (i.e. 0.9, 1, 1.25, 1.6, 2.5, 4, 6.3, 10, 16, 26 kHz) and the pulse width ranged between 10 μs and the maximum achievable at that frequency ((duty cycle: 1 / frequency—18 μs) / 2, Fig. 1A).

### Quantification of electrically-evoked neural responses

The primary outcome of interest was the ability to produce directional nerve activity, or conversely, the observed change in vagus ECAP amplitude when combining the activation stimuli with different blocking parameters. Figure 1 shows the process for determining the ECAP response, involving stimulation across the wide range of current levels available (Fig. 1B). The root-mean-square (RMS) of the ECAP within the post-stimulus window of interest (3–9 ms) was measured and compared to the stimulation current level to define the growth curve (Fig. 1C). The example shows a quantification of the growth curve of the vagal ECAP response when using a 10 kHz block with 80% duty cycle and 4 mA current (Fig. 1C). The response was fit with a logistic growth curve. The fitting curve was then used to define the saturated response, as the ECAP growth plateau or the maximum response at the limit of 2 mA when the response did not reach the plateau. The analytical ECAP threshold was then defined as the current level in the curve producing 10% of the saturation ECAP RMS (Fig. 1D).

## Statistical analysis

Differences in ECAP threshold or saturation between different frequencies and duty cycles were assessed using a repeated measures one-way ANOVA. Correlations between ECAP threshold vs. saturation or ECAP block vs. blocking charge per pulse, were assessed using a Pearson’s correlation coefficient test.. A repeated measures two-way ANOVA was used to assess differences in blocking effect when applying afferent VNS (aVNS) vs efferent VNS in either a 6- or 8-electrode array. Statistically significant differences were accepted as p < 0.05 and MATLAB software (R2022b, The MathWorks Inc., USA) used for all analysis.

## Results

### Nerve blocking by stimulation parameters

ECAPs were successfully recorded from 18 of 20 rats implanted acutely with an electrode cuff on the abdominal vagus nerve. Changes in ECAP threshold current and amplitude saturation were recorded in these afferent evoked responses when applying high-frequency blocking stimuli and/or high duty cycles (1 / frequency—18 μs) / 2, Fig. 1A) to the middle electrode pair, while recording from the rostral pair (Fig. 1C, D). Throughout these tests, a constant pulse amplitude of 4 mA was applied, which was the maximum delivered by the stimulator.

#### Blocking frequency

**Fig. 2:**
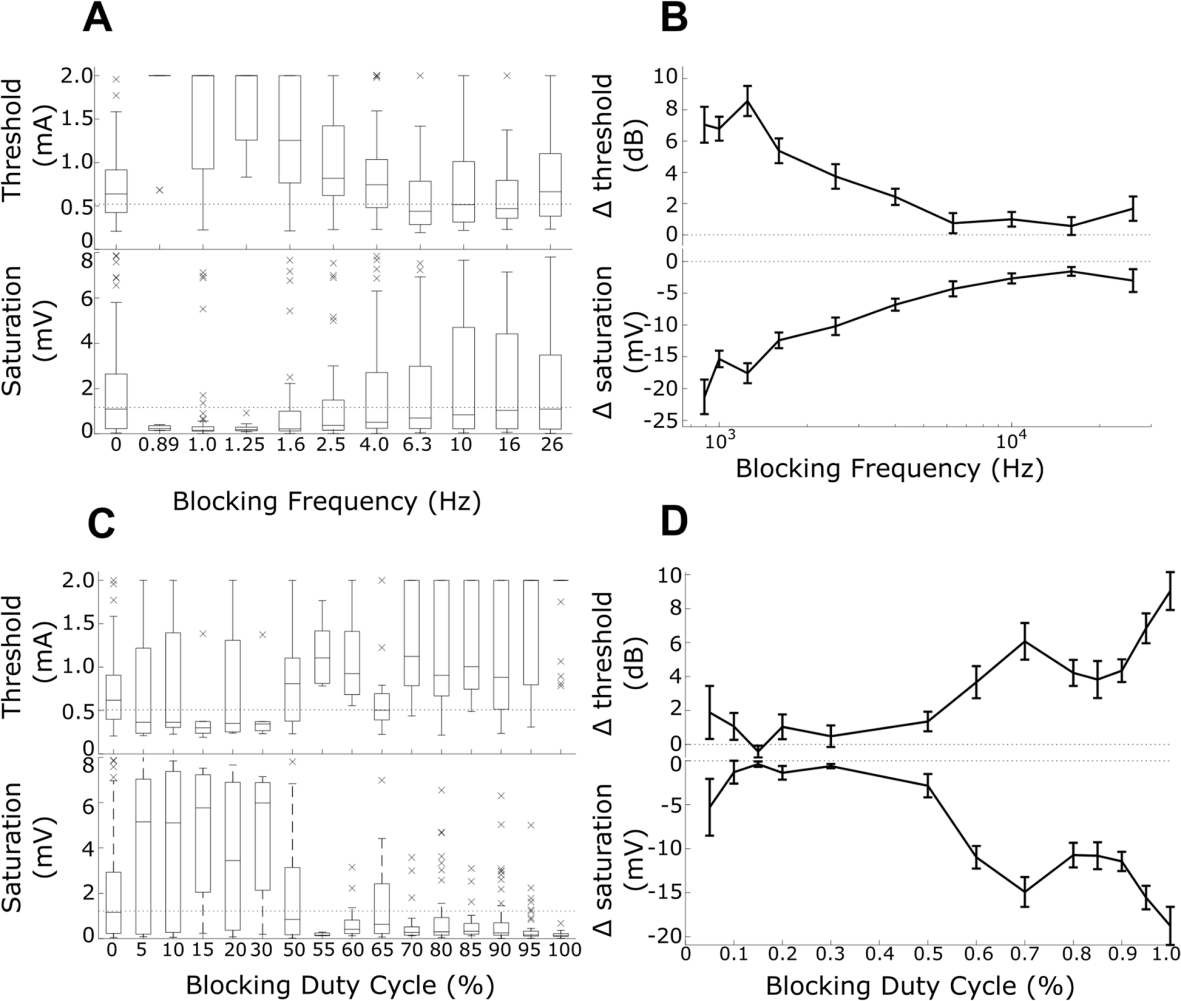
Effect of frequency and duty cycle on blocking efficacy. **A** Effect of blocking stimulation frequency on the ECAP response, showing threshold on top and saturation amplitude at the bottom. **B** Relative change with each blocking frequency vs. control ECAPs in the same experiment (mean ± std. error). The blocking effects were more evident at lower kilohertz frequencies. **C** Effect of blocking stimulus duty cycle on the ECAP response, where high duty cycles improved block. Threshold on top and saturation amplitude on the bottom. **D** Relative change with each duty cycle vs. control ECAPs in the same experiment. The blocking effects were more evident at higher (≥ 70%) duty cycles. Data in A and C show individual values (indicated by ‘x’), while data in B and D indicate mean ± SEM

Altering the frequency of stimulation resulted in a significant difference in the ECAP response (Fig. 2A, B), where lower blocking frequencies increased threshold (repeated measures ANOVA *F* = 11, *p* < 0.001) and reduced the response saturation (*F* = 4.0, *p* < 0.001).

#### Duty cycle

The duty cycle was also a significant factor in the ECAP changes observed with blocking stimulation (2C, D), where high duty cycles led to both an increase in threshold (*F* = 4.2, *p* < 0.001) and a decrease in saturation (*F* = 8.8, *p* < 0.001). A significant change in the measured signal was therefore observed for frequencies below 4 kHz and duty cycles above 60% (Fig. 2C, D).

#### Signal extraction

**Fig. 3:**
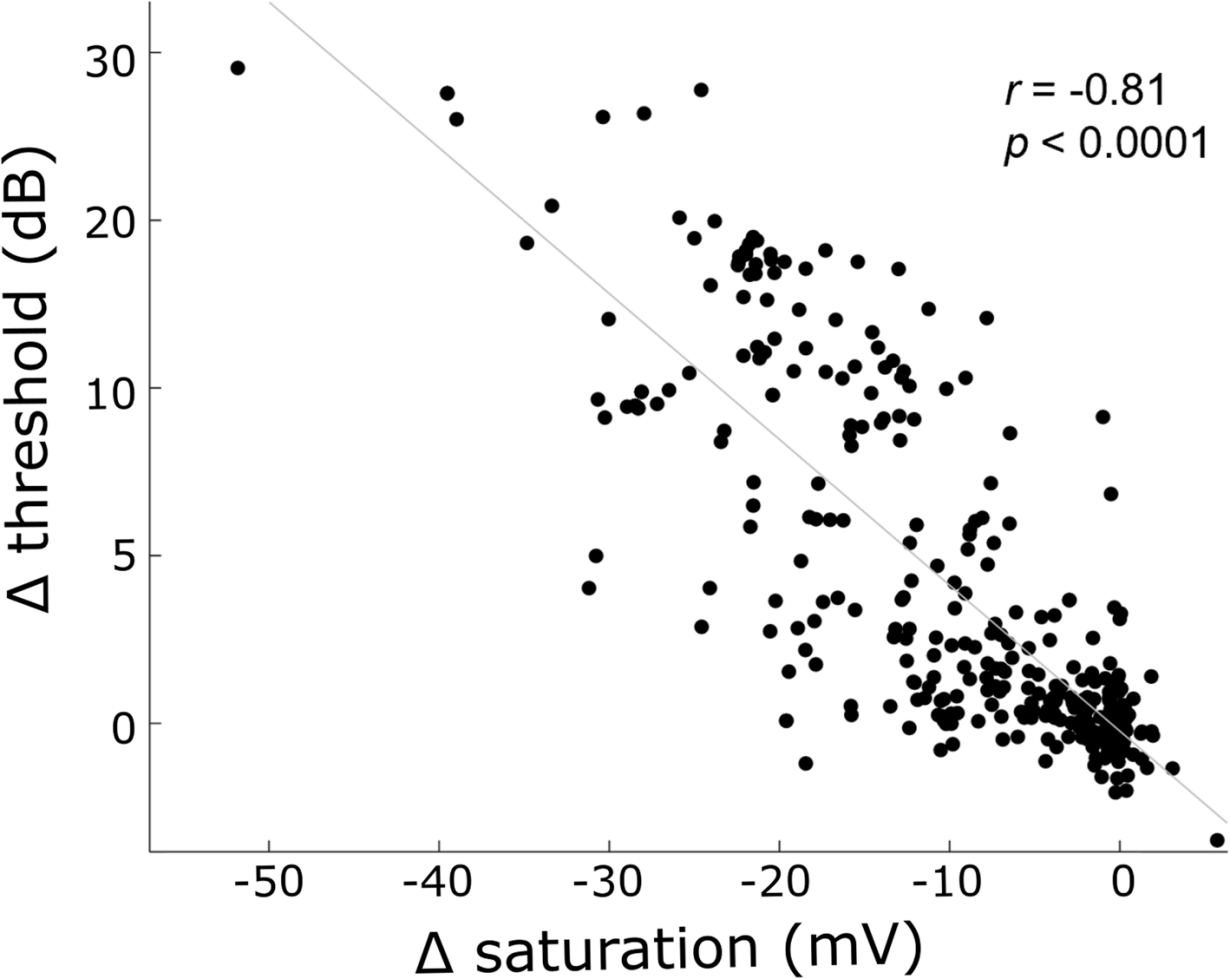
Signal extraction. Correlation of changes in ECAP threshold vs saturation amplitude, expressed as ratio to control (no-block) condition. Dots indicate data from individual recordings, while the line shows linear fit (r = -0.81, p < 0.0001)

The estimate of threshold was affected by noise levels from the blocking artifacts, as well as the accompanying decrease in ECAP amplitude saturation (including cases where saturation plateau was not reached within the 2 mA limit). Both estimations of block, i.e. threshold and saturation, were correlated when expressed as relative change from control (Fig. 3; *r* = -0.81, *p* < 0.0001). This was not unexpected due to the definition of threshold as a proportion of saturation. A principal component analysis was therefore used to compound the measure of nerve blocking and estimate the contributions of each variable to the response. The first component accounted for 94% of the variance and thus was used for subsequent analysis with weights of -0.43 for threshold and 0.91 for saturation.

#### Combining frequency and duty cycle

**Fig. 4:**
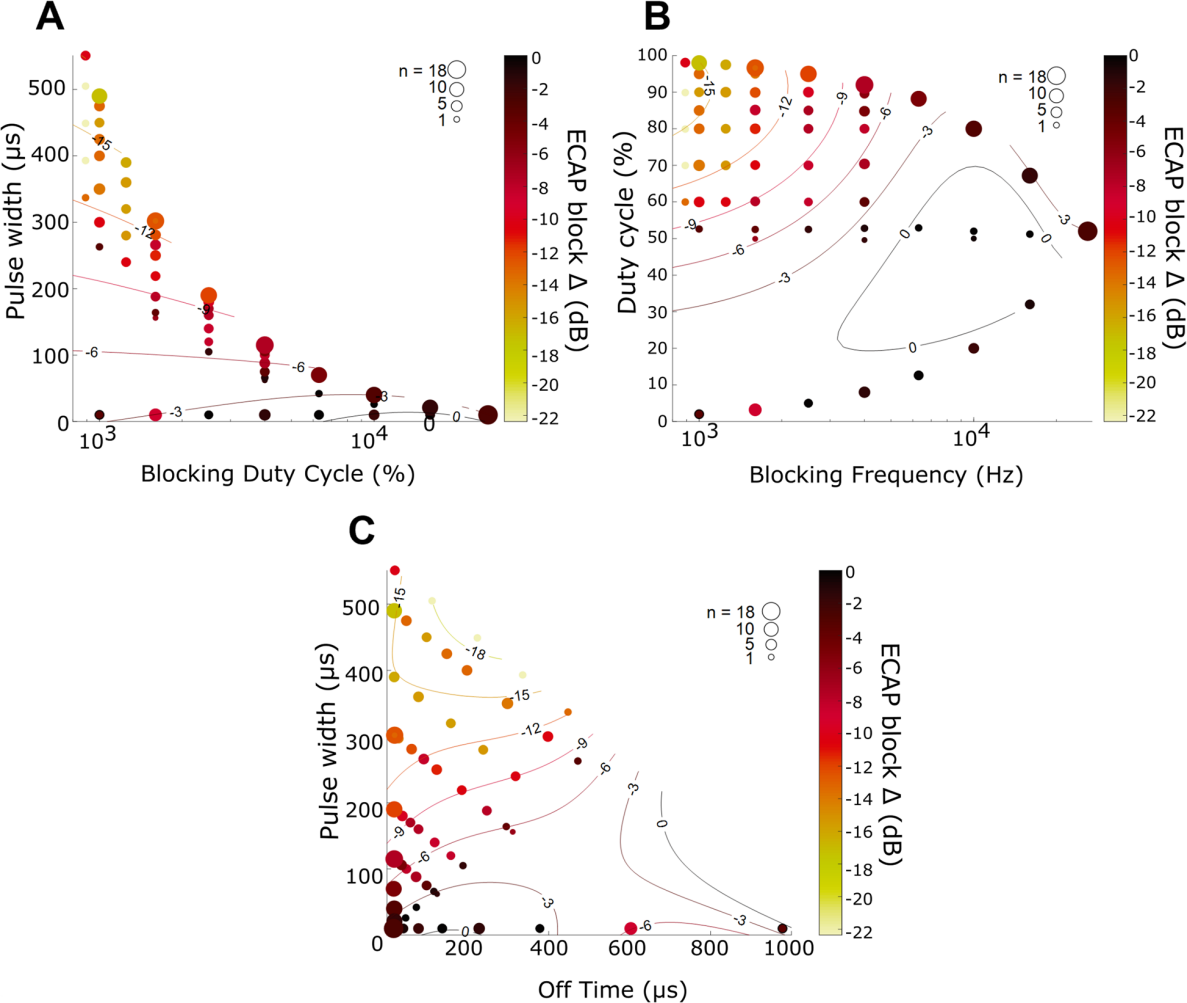
Estimation of ECAP block (change in threshold and saturation amplitude) with respect to various combinations of pulse timing parameters of the blocking stimuli. The color indicates magnitude of the change in ECAP, while the size of each circle indicates the number of animals aggregated for that parameter combination. The contour curves show a 3rd order polynomial fit as visual aid

The resulting nerve block estimate from threshold and saturation changes was compared to the timing parameters of the stimulation pulses. Figure 4 shows that the best vagus nerve block was observed when combining low frequency and long pulse width, which can also be represented as low frequency and high duty cycle. For example, a significant block of -12 dB or more can be obtained with frequencies below 2 kHz and pulse widths beyond 300 µs or duty cycle above 70%. An alternative way of studying the pulse parameters is to use the off-time or pause between pulses. The block of -12 dB thus occurs with pulse widths longer than 300 µs and an off time below 400 µs before the next pulse.

#### Regression model of stimulation parameters

The blocking stimulation timing parameters were assessed using stepwise linear regression to prioritize them by quantifying the contribution of each parameter to the variance. After controlling for individual animals, the pulse width was the parameter that explained the highest proportion of the variance (34%). The addition of duty cycle, off time and frequency to the regression model did not significantly increase the predictive power, as show in Table 1.

**Table 1:** Stepwise linear regression of ECAP block measure vs the various pulse timing parameters

Step	Predictor term	F statistic	P value	R^2^	Δ R^2^
0	Animal number	6.8	2 × 10^–14^	0.2751	0.27
1	Blocking pulse width	26.7	9 × 10^–55^	0.6135	0.34
2	Blocking duty cycle	26.1	0.11	0.6204	0.007
3	Blocking off time	25.2	0.04	0.6249	0.0045
4	Blocking frequency	24.0	0.65	0.6250	0.00003

### Effect of charge on nerve block

**Fig. 5:**
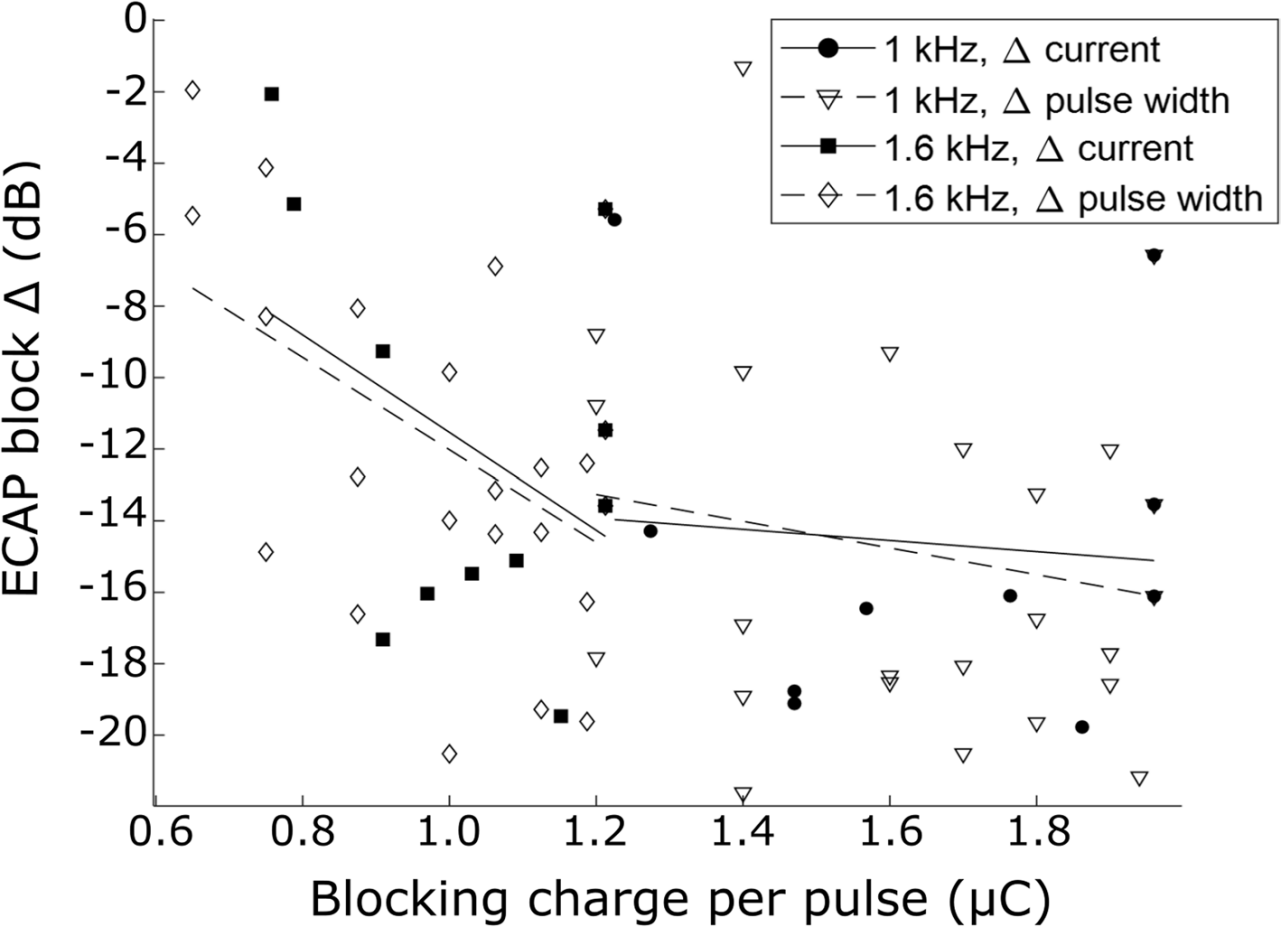
Comparison of the effect of charge per pulse on nerve block, by varying either current level or pulse width. Lines show linear fit for each stimulation mode and frequency

In a subset of experiments (*n* = 3), the effect of charge per pulse on the magnitude of blocking was estimated by varying the current. The effect of charge was assessed at the blocking frequencies of 1 kHz and 1.6 kHz using a high duty cycle above 60% (Fig. 5). The magnitude of the block depended significantly on the charge delivered per pulse (ANOVA, *p* = 0.003). With 1 kHz the block improved by -1.6 ± 6.1 dB for each µC of charge by varying current and by -3.7 ± 4.2 dB/µC by varying pulse width; while at 1.6 kHz the changes were -14 ± 10 dB/µC for varying current and -13 ± 5 dB/µC for varying pulse width. In contrast, the mode of varying the charge (constant current vs constant pulse width) was not a contributor to the effect (*p* = 0.34), nor was the effect of blocking frequency using two values (*p* = 0.23, Fig. 5).

### Spatial extent of nerve block

The focality of the blocking stimulation was assessed by determining the spatial extent of the nerve block.

**Fig. 6:**
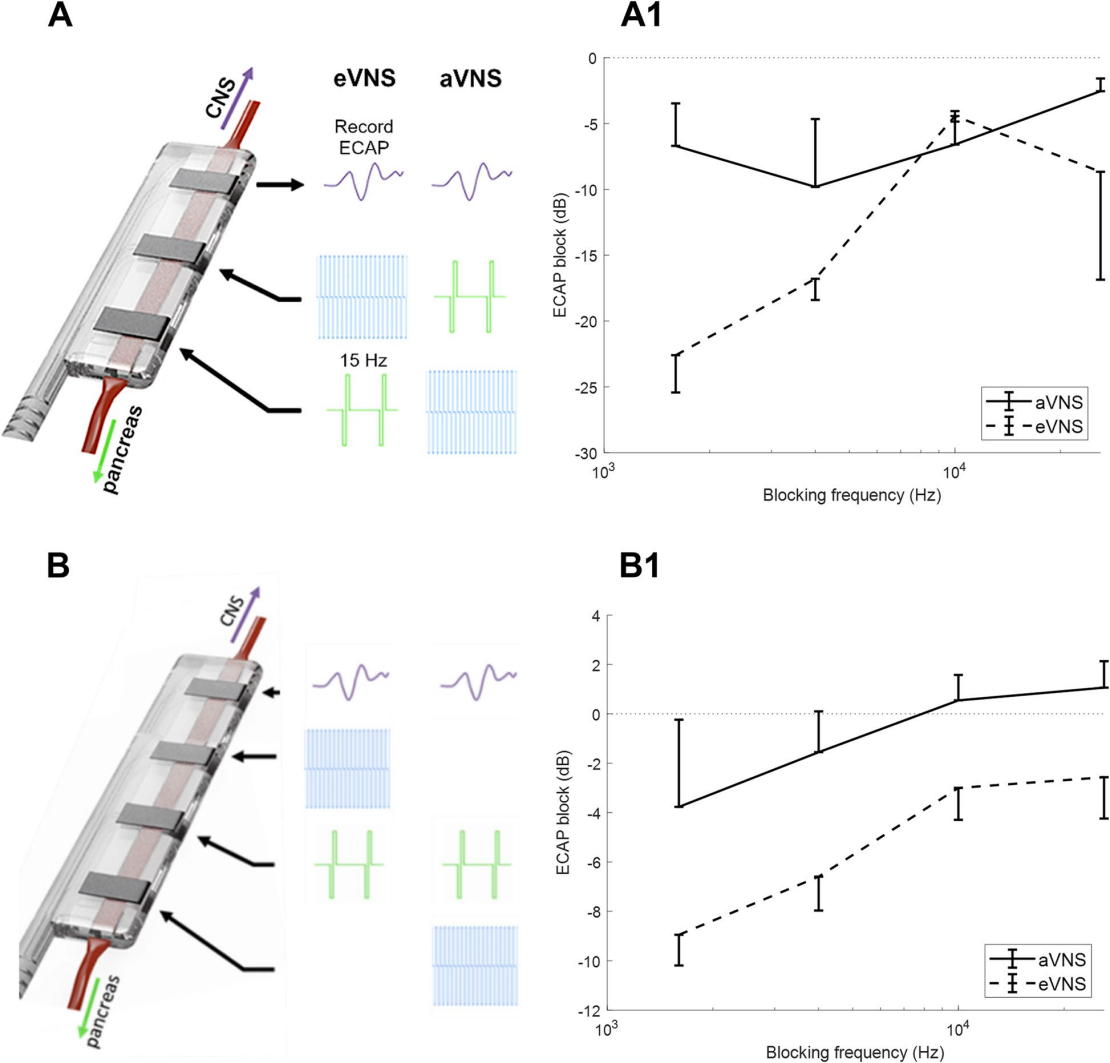
Spatial extent of the nerve block. The location of activating and blocking stimuli were exchanged so that blocking was applied to the caudal electrode pair, activating stimuli to the middle pair and recording on the rostral pair to assess the focality of the nerve. Assessment of afferent and efferent vagus nerve stimulation (aVNS, eVNS) in 6-electrode array (electrode pairs were 3.4 mm apart: (A, A1) and 8-electrode array (electrode pairs were 2.22 mm apart: B, B1) design. Data in A1 (n = 3) and B1 (n = 2) show mean ± SEM

*6-electrode array experiments*: for these experiments that used the 6-electrode array in which electrode pairs were 3.4 mm apart. The location of activating and blocking stimuli were exchanged so that blocking was applied to the caudal electrode pair, activating stimuli to the middle pair and recording on the rostral pair (as per usual, Fig. 6A). This configuration, referred to as afferent VNS (aVNS, Fig. 6A, A1) allows assessment of the ability to activate the nerve adjacent to the block. Analysis of the effectiveness of the ECAP block across increasing blocking frequencies (Fig. 6A1) shows there was a significant difference in the ECAP blocking effect when applying afferent VNS (aVNS) vs efferent VNS (eVNS, repeated measures ANOVA *F* = 7.5, *p* = 0.01, *n* = 3, Fig. 6A1). This indicates that the blocking stimuli was ‘focal’, as when blocking was applied to the caudal electrode pair there was little to no blocking effect at this location to disrupt the activation of the nerve via the middle pair (aVNS configuration).

*8-electrode array experiments:* an 8-electrode array (Fig. 6B) was subsequently developed in which electrode pairs were 2.22 mm apart. The 8-electrode array design now allowed a closer distance between the blocking and activating electrode pair (2.22 mm), thereby allowing us to assess if ECAPs could still be recorded (on the rostral pair to maintain similar recorded ECAP latencies, Fig. 6B). Although a total of *n* = 5 experiments were conducted, only *n* = 2 animals generated successful recordings due to persistent electrode breakages. Analysis of the effectiveness of the ECAP block across increasing blocking frequencies again shows there was a significant difference in the nerve blocking effect when applying eVNS vs. aVNS (*F* = 8.4, *p* = 0.01, Fig. 6B1). This confirmed that the nerve blocking stimulation levels did not prevent activation with an electrode pair at least 2.22 mm away. Finally, the blocking frequency in the eVNS configuration had a significant effect on the effectiveness of the block (*F* = 3.2, *p* = 0.02), confirming that using the 8-electrode design fielded similar results to that report with the 6-electrode design (Fig. 2A, B).

## Discussion

The use of efferent vagus nerve stimulation (eVNS) has promising glucose lowering effects in a rat model of type 2 diabetes (Payne et al. 2022). However, the blocking stimuli used in that study (26 kHz) are not compatible with commercially available implantable pulse generators and is therefore an impedance to translation. We therefore aimed to determine the range of blocking stimulation parameters (frequency and duty cycle) that generate an efficacious neural block while also residing within range of what commercial devices are able to safely deliver (≤ 10 kHz). The high-frequency blocking stimulation produced a clear and gradual change in the recorded ECAP signal of all rats, with the most significant ECAP blocking effects seen from 0.9—4 kHz, well within what can be delivered safely clinically (Shikora et al. 2013). Pulsatile stimulation is therefore able to reduce vagus nerve excitability in a manner sufficient to produce directional conduction of evoked activity. This partial block creates a window of complete evoked activity block between normal activation threshold and the increased threshold with blocking stimuli; but also diminishes the maximal or saturated evoked activity amplitude, likely a result of reducing the number of fibers available to conduct.

Most previous studies have provided insight into the effect of frequencies, waveform and current amplitude using large myelinated somatic nerve tracts as a biological platform. Effective blocking frequencies range from 200 Hz to 30 kHz with either sinusoidal or square wave functions, and amplitudes range from 0.3 to 10 mA in the in the sciatic nerve (Solomonow et al. 1983; Kilgore and Bhadra 2004a, b; Bhadra and Kilgore 2005), and pudendal nerve (Tai et al. 2004; Bhadra et al. 2006). Other types of electrical blocking strategies also exist such as DC (Kilgore and Bhadra 2004a) and monophasic high frequency (Solomonow et al. 1983), and are affective in blocking large myelinated tracts; however, they are not charge balanced and not safe for clinical use (Kilgore and Bhadra 2004a).

Here we assessed the effect of high frequency blocking on the rat abdominal vagus nerve which consists predominantly (97–99%) of unmyelinated, slow firing (0.2 – 2 m/s) C-fibers (Prechtl and Powley 1990). The parameter predictive of nerve blocking was pulse width, which was equivalent to charge per pulse given the current was usually fixed at the stimulator maximum limit of 4 mA. The second priority was maximizing duty cycle, or equivalently, minimizing off-time. However, duty cycles above ~ 80% had a largely similar effect, meaning there can be time allocated for passive charge recovery strategies such as electrode shorting without detriment. Therefore, as a rule of thumb, it should be considered that lower frequencies in the kilohertz range will produce a better nerve block by allowing for wider pulse widths; and that these must be provided with minimum off-time to maximize the duty cycle. However, lower frequencies at constant current will result in larger charge densities, so careful selection of the frequency is needed to remain within the safe stimulation limits of the electrode (Cogan et al. 2016).

With that criteria in mind, this pulsatile blocking stimulation shares the philosophy behind kilohertz-frequency-alternating-current (KHFAC) which has been shown successfully in the subdiaphragmatic vagus nerve (Waataja et al. 2011). Alternating current is thus equivalent to a bi-phasic signal with 100% duty cycle. Pulsatile stimulation will however feature a constant current (or voltage) “square” signal. Depending on the hardware implementation, a square signal could be easier to produce and perhaps better in its ability to balance injected charge between phases, while allowing passive charge recovery during a short off time where the electrodes are connected together. It is conceivable that lower frequencies that allow larger charges per phase are of similar benefit to both KHFAC and pulsatile blocking strategies.

Here we showed that the high frequency nerve block was focal and enables activation of the C-fiber efferent nerve bundle at distances less than 2 mm away. Despite growing evidence of the clinical utility of high frequency blocking, there is limited understanding of mechanisms. The most predominant theory suggests that sodium channels become inactivated, due to an increased inward sodium current, compared to the outward potassium current, leading to a forced (but reversible) sustained depolarization of unmyelinated C-fiber axonal membranes under the electrodes delivering the blocking stimulation (Kilgore and Bhadra 2014). As a result, the persistent depolarization state does not favour propagation of action potentials (i.e. the refractory period) causing a cessation of neural conduction (Tai et al. 2005).

A limitation of this study was not investigating the functional effects of optimised blocking stimuli on the glycaemic levels during a glucose challenge in an anaesthetised diabetic rat. However, future studies will investigate the efficacy of these optimised parameters (1–4 kHz, > 70% duty cycle, 4 mA) in a chronic, awake diabetic rat model as a next step to translation.

There are two applications to optimizing eVNS stimulation parameters. We have previously shown that eVNS delivered using a block at 26 kHz, 4 mA and 10 µs/phase reduces an induced glycemic response in a diabetic rat model, and could have potential application as a drug-free therapy of type 2 diabetes (Payne et al. 2022). As a second application, eVNS applied to the cervical vagus nerve reduces activation of fibers that inadvertently cause off-target effects and significantly reduces systemic inflammation in a rodent model of sepsis (Patel et al. 2017). The use of eVNS in the cervical vagus nerve to selectively activate the cholinergic anti-inflammatory pathway could have significant implications to a growing number of clinical for the for the treatment of inflammation in Crohn’s disease (ClinicalTrials.gov Identifiers, NCT02311660) (Sinniger et al. 2020) and rheumatoid arthritis (NCT04539964)(Genovese et al. 2020).

## Conclusion

Taken together, this study describes a range of high frequencies, duty cycles and currents of stimulation that generate an efficacious, focal axonal block of a predominantly C-fiber tract. These findings could potential application to support the translation of eVNS for the treatment of type 2 diabetes.

## Data Availability

The datasets supporting the conclusions of this article are available in the Dryad data repository (10.5061/dryad.gtht76hrh).

## Abbreviations

ECAP: Evoked compound action potential
KHFAC: Kilohertz frequency alternating current
DC: Direct current
ANOVA: Analysis of variance
eVNS: Efferent vagus nerve stimulation
RMS: Root-mean-square
